# Rodents’ responses to manipulated plant litter and seed densities: implications for restoration

**DOI:** 10.7717/peerj.9465

**Published:** 2020-07-06

**Authors:** Nancy Nicolai

**Affiliations:** Department of Biology, University of New Mexico, Albuquerque, NM, USA

**Keywords:** Small mammal, *Dipodomys spectabilis*, *Perognathus flavus*, *Onychomys leucogaster*, Wildlife habitat, Grassland, Plant litter, Food availability

## Abstract

Rodent populations in arid grasslands do not always track seed production, possibly due to high levels of plant litter. When natural disturbances are suppressed, litter accumulates becoming physically complex, causing rodents to harvest fewer seeds per equivalent time foraging. It also alters security from predation. Restoring natural disturbances may be an important element in conserving rodent communities. The aim of this study was to assess the influence of two levels of plant litter cover and seed densities on nocturnal rodent population characteristics in a semiarid grassland. Specifically, I hypothesized that kangaroo rats, pocket mice, grasshopper mice, and total rodents would be higher in the sparse plant litter treatment than dense litter, whereas deer mice would be lower in sparse plots. I further hypothesized that kangaroo rats and deer mice would be higher in the seed augmented treatment compared to the unseeded treatment. A prescribed fire removed litter in four of eight plots prior to sowing native seeds 1 year postfire into two burned and two unburned plots. Rodents were live-trapped during spring and fall 1 year. Sparse litter treatment had higher total rodent abundance, biomass, and frequency of offspring compared to dense plots indicating use of stored seeds. Banner-tailed kangaroo rats had higher abundance, implying reduced predation risk. Pocket mice body mass was greater in dense plots. After winter, seeded plots had higher kangaroo rat body mass and grasshopper mice abundance than unseeded, reflecting the use of stored seeds. These short term results demonstrate litter’s physical complexity may be equivalent to seed pulses on the responses of nocturnal rodents. Managers might positively influence grassland rodents by providing a mosaic of varying levels of plant litter.

## Introduction

Small mammal populations in aridlands are regulated by resource pulses which supply food and habitat (Brown, Reichman & Davidson, 1979). Sometimes, however, populations do not grow following pulses (Baez et al., 2006; Shenbrot, 2014). High levels of plant litter alter population feedback to resource pulses (Shenbrot, 2014). When grasslands lack natural disturbances (sensu Paine (1966) and Sousa (1984)), plant litter accumulates causing rodents to forage inefficiently (harvest fewer seeds or gather fewer nutrients per equivalent time foraging) (Reed, Kaufman & Kaufman, 2006). Grassland rodents’ security from predation (predation risk) is also altered by plant litter (Clark & Kaufman, 1991; Longland & Price, 1991). Accumulated litter lowers rodent density and diversity (Abramsky, 1978; McCarty, 1978; Thompson & Gese, 2013) and may reduce a keystone species (Hoffmeister, 1986). A large-scale disturbance can decrease litter which may alter rodent abundance by allowing foragers to gather food more efficiently and detect and escape predators. Restoring natural disturbances may be an important element in conserving rodent communities.

In North American grasslands, plant litter is a prevalent component of rodents’ habitats. Senescent plants generate litter accumulating yearly. Prior to European settlement, losses regularly occurred from decomposition, fire, and herbivory. Suppression of fire and demise of bison (*Bison bison*) and prairie dogs (*Cynomys*) contribute to high amounts of senescent litter (Agnew, Uresk & Hansen, 1986; Fuhlendorf et al., 2012). This accumulated litter is negatively related to rodent mean body mass, diversity, richness, and total rodent biomass in a northern shortgrass prairie (Thompson & Gese, 2013). Forager efficiency is reduced when rodents move through the complex architecture of standing and downed litter and when they pursue post-dispersal seeds bound in litter (Reed, Kaufman & Kaufman, 2006; Thompson & Gese, 2013). Litter can entrap herbaceous seeds and prevent them from reaching the ground (Donath & Eckstein, 2010; Rotundo & Aguiar, 2005) where rodents forage. For example, when dense, it can take 1 month for grass seeds to reach the soil surface after they are dispersed (Fowler, 1986). Thus, foraging efficiency is reduced because rodents collect fewer seeds as litter depth and biomass increases (Reed, Kaufman & Kaufman, 2006).

Litter’s physical architecture is an important factor in predation risk: risk increases for species that attempt to elude predators in simply structured vegetation and litter (Reichman & Price, 1993), or decreases when hiding places from predators are available (Clark & Kaufman, 1991). Reichman & Price (1993) concluded that desert Heteromyidae with bipedal, saltatorial locomotion chose habitats with sparse vegetation and litter where they can see and hear better than in denser vegetation and litter. Further, hopping allows them to escape owl predation more frequently in sparse areas than quadrupeds (Longland & Price, 1991). In contrast, moderate amounts of grassland litter may provide security from predators. Nocturnal rodents typically reduce activity during the bright light of a full moon when they are at greater risk from predation (Clark & Kaufman, 1991; Lockard & Owings, 1974). On dark nights male deer mice (*Peromyscus maniculatus* Wagner) spend more time foraging for barley seeds in sparse amounts of grassland litter (26 g/m^2^) compared to moderate (400 g/m^2^), but on bright nights they forage longer in moderate than sparse (Clark & Kaufman, 1991).

Natural disturbances can change the biomass of accumulated litter. The first year after fire, for example, there is a decrease in total above-ground plant biomass in semiarid grasslands (Brockway, Gatewood & Paris, 2002; Collins et al., 2017; Scheintaub et al., 2009). As a result, perennial dicot productivity (Parmenter, 2008) and herbaceous diversity increases (Ladwig et al., 2014). By the second year, plant biomass is greater with exact amounts depending upon seasonal soil moisture (Mulhouse, Hallett & Collins, 2017). However, the number of seed inflorescences is similar between burned and unburned herbaceous vegetation during 2 years postfire (Nicolai, 2019). Populations of rodent species are either unchanged or recover in 1 year (Letnic, Tischler & Gordon, 2013; Plavsic, 2014).

Yearly plant production in North American grasslands is the resource pulse fundamental to maintaining herbivore productivity and density (Lightfoot et al., 2012; Milchunas & Lauenroth, 1993). This productivity includes the generation of post-dispersal seeds. Rodents in arid and semiarid grasslands commonly depend on consumption of dispersed seeds as their main diet year round or during the winter (Brown, Reichman & Davidson, 1979; Genoways & Brown, 1993; Grant & Birney, 1979). Even carnivorous grasshopper mice (*Onychomys*) will switch to seeds in winter if prey abundance is low (Jahoda, 1970). Elevated seed consumption improves population characteristics such as mean reproductive output and mean pup body mass (Parsons et al., 2005; Soholt, 1977). Additionally, seeds with high water content and nutritional constituents are known to improve basic physiological needs (Dearing, Mangione & Karasov, 2000) such as water balance (Frank, 1988). Multiple nocturnal rodent taxa respond differentially to varying levels of litter and seeds in southern grasslands (Abramsky, 1978; Duval, Jackson & Whitford, 2005; Grant & Birney, 1979; Stapp, 1997a). Typical Heteromyidae include the bipedal banner-tailed kangaroo rat (*Dipodomys spectabilis* Merriam) and Ord’s kangaroo rat (*D. ordii* Woodhouse) along with the quadrupedal silky pocket mouse (*Perognathus flavus* Baird). All are granivores; they use a complex series of seasonal foraging behaviors to collect grass and forb seeds which they transport to their underground nests for storage and from which they obtain nourishment throughout the year (Best, 1988; Best & Skupski, 1994; Hope & Parmenter, 2007; White & Geluso, 2012; Wolff & Bateman, 1978). Kangaroo rats also store seeds in scattered caches throughout their home ranges. The addition of commercial seeds in their habitats will increase population characteristics of banner-tailed kangaroo rats, such as the number of reproductive individuals, but silky pocket mice are unchanged (Brown & Munger, 1985; Edelman, 2011). Banner-tailed kangaroo rats occupy desert grasslands where grasses are short and sparse (Hoffmeister, 1986).

A grassland’s physical architecture may be equivalent to resource pulses, shaping abundance and population characteristics of small mammals. In shortgrass prairie, Ord’s kangaroo rats and silky pocket mice densities are positively correlated with bare ground and low litter, respectively (Thompson & Gese, 2013). Cricetidae is represented by deer mice taxa (*Peromyscus*), one or two species of grasshopper mice (*Onychomys*) and two woodrats (*Neotoma*). Generally, grassland deer mice consume seeds and arthropods matching seasonal availability (Hope & Parmenter, 2007), and they prefer moderate litter cover to escape predators (Clark & Kaufman, 1991). Grasshopper mice are carnivorous, consuming seeds during fall and winter (Hope & Parmenter, 2007). Northern grasshopper mice (*Onychomys leucogaster* Wied-Neuwied) store food including seeds in their nest and food burrows (Jahoda, 1970; Ruffer, 1965). Although grasshopper mice prefer habitats with higher insect abundance (Stapp, 1997b), they are also documented more often in low litter cover than high cover (McCarty, 1978; Rebar & Conley, 1983). Woodrat density in desert grassland is limited by the number of large shrubs available for their nests (Whitford, 1976), but not litter cover (Jones, Bock & Bock, 2003).

Non-colonial, nocturnal rodents are key components in food webs of arid and semiarid grasslands. They influence vegetation abundance and diversity by many mechanisms, such as herbivory (Maron & Kaufman, 2006; Rebollo et al., 2013), post-dispersal seed predation (Bricker, Pearson & Maron, 2010; Louda, 1989; Marshall & Jain, 1970; Pulliam & Brand, 1975), grass competitive release (Martinsen, Cushman & Whitham, 1990; Nicolai, 2019) and soil engineering (Fields, Coffin & Gosz, 1999; Nicolai, 2019). Further, they alter predator density. Understanding nocturnal rodent responses to varying levels of plant litter and seed abundance improves our ability to determine an ecosystem’s potential consequences from alterations to disturbances including fire, mammalian herbivory, and extremes in precipitation. The aim of this study was to assess the influence of two levels of litter cover and seed density on each species’ population characteristics, rodent community abundance and rodent biomass in a semiarid grassland during 1 year. A species’ population characteristics included abundance, individual body mass, frequency of males’ and females’ reproductive classes, and numbers of offspring. Specifically, I hypothesized that (1) the population characteristics of kangaroo rats, pocket mice, grasshopper mice and the abundance of the rodent community would be higher in the sparse litter cover treatment than dense litter, whereas (2) deer mice would be lower in sparse compared to dense litter cover treatment. I further hypothesized that (3) the population characteristics of kangaroo rats and deer mice would be higher in the seed augmented treatment compared to the unseeded treatment.

## Materials and Methods

### Study area

Research was conducted at Sevilleta National Wildlife Refuge (Sevillta NWR), Rio Grande Valley, New Mexico, USA (elevation 1,600 m a.s.l.; 34°20′30″N, 106°43′30″W). Sevilleta NWR grasslands stand at an ecotone between US Geological Survey’s (1996) Chihuahuan Foothill-Piedmont Desert Grassland and Short Grass Steppe. Plants in the study area were from both grasslands. Vegetation in the study area is a patchwork of bare ground and perennial, C_4_ warm-season grasses dominated by black grama (*Bouteloua eriopoda* (Torr.) Torr) and blue grama (*B. gracilis* (Willd. ex Kunth) Lag. ex Griffiths) with approximately 40 forb species. Mean bare ground cover is 22% (SD 17%) and mean plant litter cover is approximately 50% (preliminary observations). Shrubs make up 5% aerial cover and include Torrey’s jointfir (*Ephedra torreyana* S. Watson) and fourwing saltbush (*Atriplex canescens* (Pursh) Nutt.). Seed density in the soil seed bank is lower than in Short Grass Steppe and similar to Chihuahuan Desert Grassland (Moreno-De Las Haras, Turnbull & Wainwright, 2016). Cattle were removed in 1975 following approximately 100 years of continuous livestock grazing. Gunnison’s prairie dogs (*Cynomys gunnisoni*) were extirpated by rangeland pest control agents in 1972 and reintroductions by Sevilleta NWR began in 1997. From 1976 to 1996 the cover of plant litter increased significantly without these herbivores (Ryerson & Parmenter, 2001). The study area is characterized by a gentle 4–5%, south-facing slope. Soils are Turney loam, a well-drained Aridisol with loam about 10 cm deep overlaying sandy clay loam to clay loam (Buxbaum & Vanderbilt, 2007; Johnson, 1988).

The climate is semiarid, mid-elevation continental with two growing seasons: cool (December–March) and warm (July–September). Mean annual precipitation (1989–2014) has been 223.9 mm yr^−1^ (MET-40 Station within 5 km of the study area (Moore, 2015)). Mean daily temperatures have been 25.3 °C in July and 2.4 °C in December (1989–2014). Total cool-season precipitation leads to shrub and most forb growth (Muldavin et al., 2008; Mulhouse, Hallett & Collins, 2017; Robinson et al., 2013) with seed production in April and May. Total warm-season precipitation leads to growth and reproduction by most perennial grasses and a different suite of forb species from August through October. Station records (Moore, 2015) showed that when treatments were initiated, total cool-season precipitation was 63% lower than the long-term average and warm-season was 18% lower. During small mammal sampling the following year, both seasons were dry at 64% and 50% lower than average during the cool- and warm-seasons, respectively.

The nocturnal rodent community in Sevillta NWR grasslands is a combination of species from Chihuahuan Foothill-Piedmont Desert Grassland and Short Grass Steppe. Generally, the community is characterized by banner-tailed kangaroo rats, Ord’s kangaroo rats, Mearn’s grasshopper mice (*Onychomys arenicola* Mearns), and silky pocket mice ((Newsome, 2017), Five-Points grassland, 1989–2012). Additionally, 15 other rarely-occurring species have been captured including mice taxa (*Peromyscus*), southern plains woodrat (*Neotoma micropus* Baird), and white-throated woodrat (*N. albigula* Hartley). At the study area, the diurnal spotted ground squirrel (*Spermophilus spilosoma*) occurred at low densities. Gophers (*Thomomys*) were present and locally active. I used Mammal Species of the World (Wilson & Reader, 2005) nomenclature for small-mammal taxa.

### Experimental design

To assess rodent population characteristics among treatments, I recorded animals from live-traps in a manipulated field study. The experiment measured the individual and combined effects of two litter and two seed treatments: a 2 × 2 factorial treatment structure in a randomized design. Four study plots were each placed randomly in a burned (sparse litter treatment) and unburned (dense litter treatment) area, making a total of eight plots. Unburned plots were adjacent to the burn area. I randomly chose two sparse and two dense litter plots for seed augmentation (four seeded treatment plots altogether) while the remaining two sparse and two dense litter plots were left as the four unseeded treatment plots. The plots measured 100 × 50 m (0.5-ha area) and were marked at each corner and were placed 100 m from each other to reduce environmental heterogeneity among plots. A study in this grassland found that not any individually marked, ear-tagged rodents were recaptured in plots that were are at least 100 m apart (A. Elliott, 2014, unpublished data, fws.gov/refuge/Sevilleta, Sevilleta National Wildlife Refuge, P.O. Box 1248, Socorro, NM 87801, USA).

### Litter treatments

A prescribed fire, May 2015, was used to create the sparse litter treatment, reducing plant litter in approximately 30 ha. When seed treatments were implemented in October 2015, plant litter had increased and consisted of leaves from herbaceous vegetation that had grown and then senesced since the fire in addition to burned stubble. Following the first year after fire at Sevilleta NWR, there could be changes to vegetation and rodents; I also waited 1 year to begin rodent sampling in order to reduce initial responses.

The dense litter treatment was unburned grassland and consisted of approximately 80% standing litter: senescent grass stems and leaves averaging 0.5 m tall, and 20% stems and woody material lying loose on the ground. The level of litter cover corresponds to typically fire-suppressed (>10 years) and lightly grazed semiarid grassland at Sevilleta NWR.

I estimated litter cover on all plots at the same time seed treatments were inaugurated, 16 October 2015, to quantify any dissimilarity between litter treatments. Twenty random samples were collected from each plot using a circular hoop measuring 0.86 diameter (0.58 m^2^-area). Estimate of litter was measured by visually estimating coverage from the top of the litter vertically and from fallen litter horizontally, both projecting on the ground (Bonham, 1989). Estimates are expressed as a percentage of the ground surface covered by combined standing and fallen litter in each hoop (Bonham, 1989; Whittaker, 1965).

I collected litter biomass in each plot on 2 and 6 November 2016 in randomly selected thick litter patches. Ten samples were gathered in each plot by cutting all plant material within a 25 × 21 cm quadrat to 2.0 cm above ground. Dry weights were obtained by placing each sample in a paper bag, oven drying for 72 h at 68 °C, then immediately weighing it using a Mettler PM 4800 Scale to 0.01 g. Litter biomass was standardized to g/m^−1^.

### Seed treatments

To assess the outcome of seed augmentation on the rodent community, naturally occurring species were hand sown at double the seed density recorded for the grassland seedbank at Sevilleta NWR (Koontz & Simpson, 2010). Therefore, approximately 17,000,000 seeds were added to each seeded plot. Seed augmentation in burned plots was assumed to have similar seed densities as unburned plots because no increases in herbaceous seed inflorescences had occurred during 2 years postfire (Nicolai, 2019). I chose a seed mixture from native species that are known to be collected and eaten by dominant rodents in the study area (Hope & Parmenter, 2007). Further, all species in the seed mixture grow in the study plots. The seed mixture consisted of two grass species, blue grama and sand dropseed (*Sporobolus cryptandrus* (Torr.) A. Gray) and one forb, scarlet globemallow (*Sphaeralcea coccinea* (Nutt.) Rydb.) purchased from Chihuahuan grassland sources. A seed was the entire propagule: a grass spikelet including florets, glumes, and awns, and a forb fruit containing its seeds. The mean (*n* = 20 propagules) of one propagule’s mass was: blue grama 0.08 mg (SE 0.04), sand dropseed 0.01 mg (SE 0.01), and scarlet globemallow 0.20 mg (SE 0.20).

Seeds were sown along one 112 m transect in each seeded treatment plot by walking from one corner of the plot to the other. One clump of approximately 3,300 propagules was placed on the soil surface every 20 m along the transect in 3 × 3 cm patches. This length and pattern were needed to assure an even spread of seeds over the entire plot making them available to many rodents. I walked the same transect without sowing seeds in each unseeded plot, thus similarly disturbing habitat in all plots. Seeds were sown during natural dispersal: 16 October 2015, at the end of the warm-season; 26 May 2016, peak of the cool-season; and 4 August 2016, peak of the warm-season.

To verify that rodents found and harvested sown seeds and that there was similar harvest probability among the eight plots, I recorded the percent of sown propagules harvested by rodents from clumps in each plot. One clump (3,300 propagules) was placed into a 8.5 cm diameter, 0.2 L paper cup weighted with a rock so it would not blow away. Cups were used to reduce seed loss from wind and were placed sideways on the soil surface so that rodents had easy access to the clump. Fourteen cups were randomly placed along one meandering 112 m-long transect in seeded plots and 29 cups in unseeded plots. Additional cups were added to unseeded plots to increase the probability that rodents would find the clumps. Clumps were offered for 19 days beginning 16 October 2015, the same day propagules were first sown into plots for the experiment. Data were the percent of cups with missing seeds and the percent of seeds removed from a cup.

### Rodent trapping

I sampled nocturnal rodents using mark-recapture live-trapping for 1 year. The short study was conducted to take advantage of litter loss after fire and before litter accumulates. Twenty-four Sherman traps (9 × 9 × 23 cm) were placed along three transects at 10-m intervals within each plot. Transects were spaced 10-m apart and were centered in the plot to reduce edge effects. Traps were baited with a mix of sunflower, millet, corn, chopped peanuts, and rolled oats. Bait was microwaved 1 min per 1 kg seeds to eliminate germination. In each trap, a ball of 100% cotton was provided for insulation. Traps were set at dusk and checked the following morning after first light. Individuals were released at capture location. Each individual was marked with ink on its chin and chest. Ink remains for over 4 days (personal observation). Individuals were each weighed and identified as to species, age (adult, juvenile, subadult), sex, and reproductive condition. I classified adult males as having fully scrotal testes or reproductively inactive and adult females as having swollen vulva (estrus), elongated nipples (lactating), embryos palpable (pregnant), or reproductively inactive. Estimates of abundance were the total number of unique individuals captured during a trapping period. Rodent biomass was calculated for each plot by summing all unique individuals’ body masses and was standardized to g/ha^−1^.

By trapping in the cool- and warm-seasons, I was able to test rodent responses to treatments over time periods that differed in rainfall, temperature, food availability, and life cycle stages of the rodents. I also took advantage of trapping after the two natural seed dispersal periods. Additional sampling periods were avoided because they may increase stress on individuals from frequent handling and increase trap attractiveness to predators, thus altering predation risk. Data were collected during two trapping periods: May 2016, 7 months after the fall seed addition and 1 year after litter reduction (prescribed burn); and October 2016, after two more seed additions were applied during the summer. And samples were collected in May to enumerate winter survivors and spring reproduction (Best & Skupski, 1994; Davies & Schmidly, 1994; Edelman, 2014; Matsuzaki & Matsuzaki, 1994) and in October to enumerate summer survivors and subadults. Four randomly selected plots were trapped 5–7 May and the remaining four plots 11–13 May. The second trapping was conducted in four randomly selected plots 27–29 October and the remaining four plots 3, 7, and 8 November. All trapping was conducted during the week of a new moon. Rodents were live-trapped safely and in accordance with American Society of Mammalogists guidelines (Gannon & Sikes, 2007). This research was conducted under a research permit from the Sevilleta National Wildlife Refuge (Special Use Permit No.10-032).

### Statistical analysis

A two-way completely randomized design ANOVA was conducted to test differences among treatment means and their interactions. Litter biomass was analyzed as a covariate when the correlation between it and the dependent variable was significant. Separate analyses were conducted for each sampling period to reduce variances caused by different rodent reproductive seasons and multiple seed applications. For each species, abundance and adult body mass were analyzed separately. Total rodent biomass, total abundance and total offspring abundance were also analyzed separately. When abundance, adult body mass, and rodent biomass data were non-normal and variances were unequal, data were transformed for analyses. Some distributions of data were highly skewed even after transformations, therefore nonparametric ANOVA (Kruskal–Wallis) was used (Zar, 2014). Litter biomass was tested using the Ryan–Einot–Gabriel–Welsch test. Chi Square Test of Association was used to compare treatments with the frequency of age classes, sexes, and categories of adult reproductive classes for each species. If expected counts for a category were below five, then two or more categories were combined (Zar, 2014). Fisher’s Exact Test was used when expected counts for a combined category were still less than five.

Significance for each analysis was at *p* < 0.05 alpha level. Analyses were performed using SPSS version 24 (SPSS Statistics, IBM, Ireland).

## Results

A total of 54 individuals in May and 55 individuals in October from seven species were caught during 1,152 trap-nights (Table 1). Banner-tailed kangaroo rats, silky pocket mice, northern grasshopper mice, and Mearn’s grasshopper mice were found in each treatment combination and were used for analyses of individual species. Generally, abundance of these species in unburned, unseeded plots was two times higher than their average long-term abundance in Sevilleta NWR grasslands ((Newsome, 2017), Five-Points grassland, 1989–2012). Mean recaptures for kangaroo rats (45% captures), northern grasshopper mice (33% captures), and Mearn’s grasshopper mice (36% captures) indicate estimates of population numbers were close to the plots’ actual abundances, but mean pocket mice recapture was 16%, a poor estimate. Southern plains woodrats and white-throated woodrats were captured in one plot next to 1.5-m tall shrubs. Mice taxa (*Peromyscus*) were not captured.

**Table 1:** Abundance of rodents captured. Numbers of nocturnal rodents in litter and seed treatments at Sevilleta National Wildlife Refuge, New Mexico, USA. Numbers are unique captures in a 3-day sampling period 5–7, 11–13 May 2016 and 27–29 October, 3, 7, and 8 November 2016.

Species	May				October			
	Dense litter		Sparse litter		Dense litter		Sparse litter	
	Unseed	Seed	Unseed	Seed	Unseed	Seed	Unseed	Seed
Silky pocket mice (*Perognathus flavus*)	8	2	5	6	3	5	3	6
Banner-tailed kangaroo rats (*Dipodomys spectabilis*)	4	0	9	9	2	2	10	11
Northern grasshopper mice (*Onychomys leucogaster*)	2	0	2	4	2	0	1	1
Mearn’s grasshopper mice (*Onychomys arenicola*)	0	0	0	0	1	1	3	2
Southern plains woodrats (*Neotoma micropus*)	1	0	0	0	1	0	0	0
White-throated woodrats (*Neotoma albigula*)	1	0	0	0	1	0	0	0
Plains pocket mice (*Perognathus flavescens*)	0	1	0	0	0	0	0	0

Female-biased sex ratios were found in kangaroo rats 2.5:1 and pocket mice 2.5:1, whereas male-biased sex ratios were observed in northern grasshopper mice 2:1 and Mearn’s grasshopper mice 2.5:1. The percent of reproductive individuals of kangaroo rats in May was 62.5% and in October was 27.5%. Both seasons the percent of reproductive pocket mice was 45.0% and of the reproducing females 43.7% were pregnant or lactating. The percent of reproductive northern grasshopper mice in May was 28.6%, however a lactating and a pregnant female were captured both seasons. The percent of reproductive Mearn’s grasshopper mice in October (the only season they were captured) was 40% including a lactating and a pregnant female. Nevertheless, very few offspring, two juveniles and six subadults, were counted from all rodent species.

### Validation of treatments

**Figure 1:**
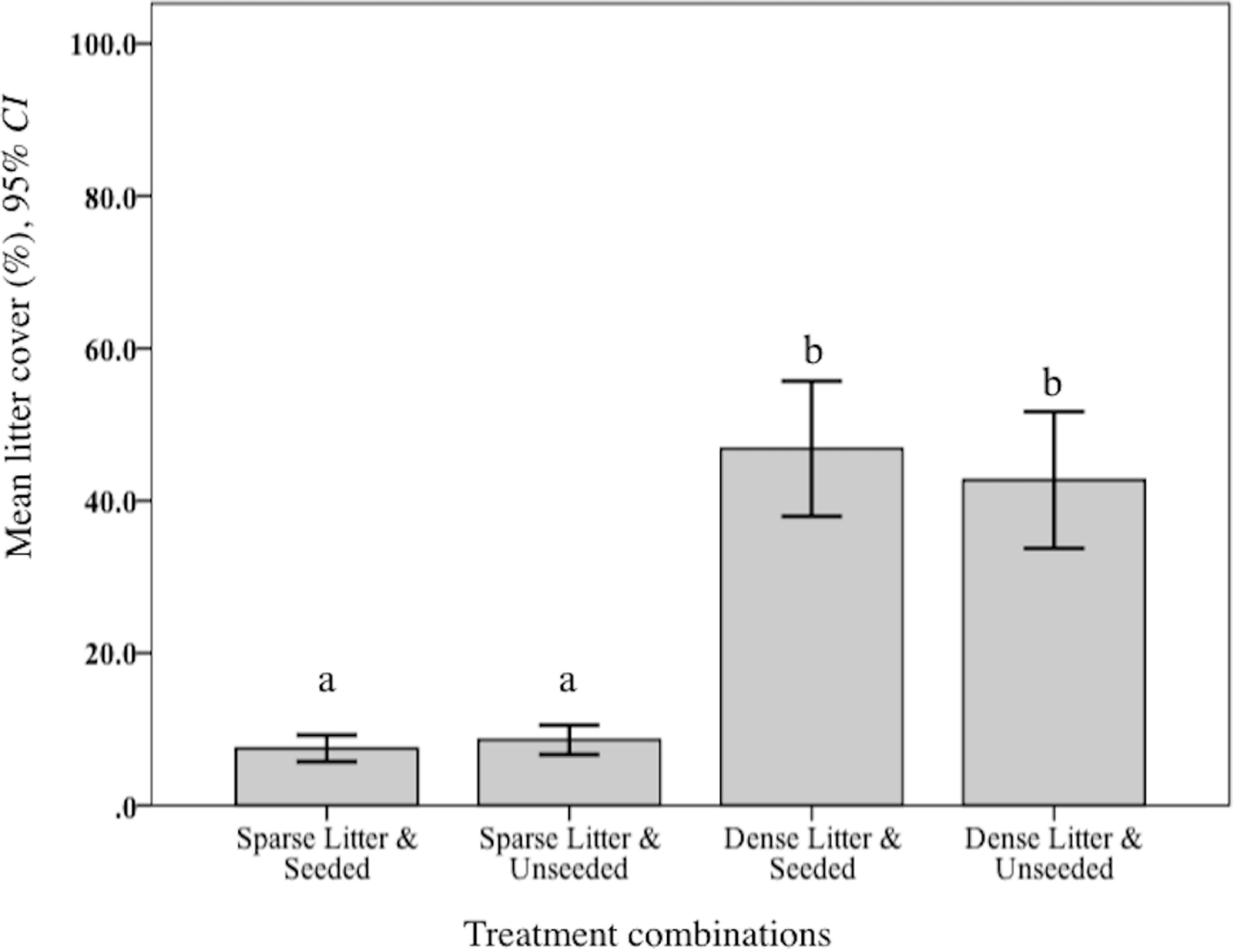
Mean litter cover at sparse and dense litter treatments. Mean (95% CI) percent litter cover at sparse and dense litter treatments 16 October 2015 where plots were seeded or unseeded, Sevilleta National Wildlife Refuge, New Mexico, USA. Post-hoc contrasts conducted using Ryan–Einot–Gabriel–Welsch test for unequal sample sizes. Means with different letters are significantly different at *p* < 0.05. *n* = 33 samples sparse-seeded plots, 39 sparse-unseeded, 40 dense-seeded, and 38 dense-unseeded.

Levels of plant litter were validated. As expected, mean percent litter cover was significantly lower in the sparse treatment compared to dense (*F*_2, 150_ = 520.09, *p* < 0.001; Fig. 1). Further, observations of cover remained widely different during the following year when rodents were sampled. Mean litter biomass varied significantly among treatment combinations (*F*_3, 48_ = 646.70, *p* < 0.001). Rodent captures were considerably lower in two dense litter plots (4.5% of total catch) and these two seeded plots had significantly greater mean litter biomass (265.5 g/m^−1^, SD 102.7 g/m^−1^) compared to the remaining two unseeded dense litter plots (171.4 g/m^−1^, SD 62.3 g/m^−1^; post hoc Ryan–Einot–abriel–Welsch *T*_3,48_, *p* = 0.05). Sparse plots, in comparison, were significantly lower than dense (69.5 g/m^2^, SD 21.0 g/m^2^).

**Figure 2:**
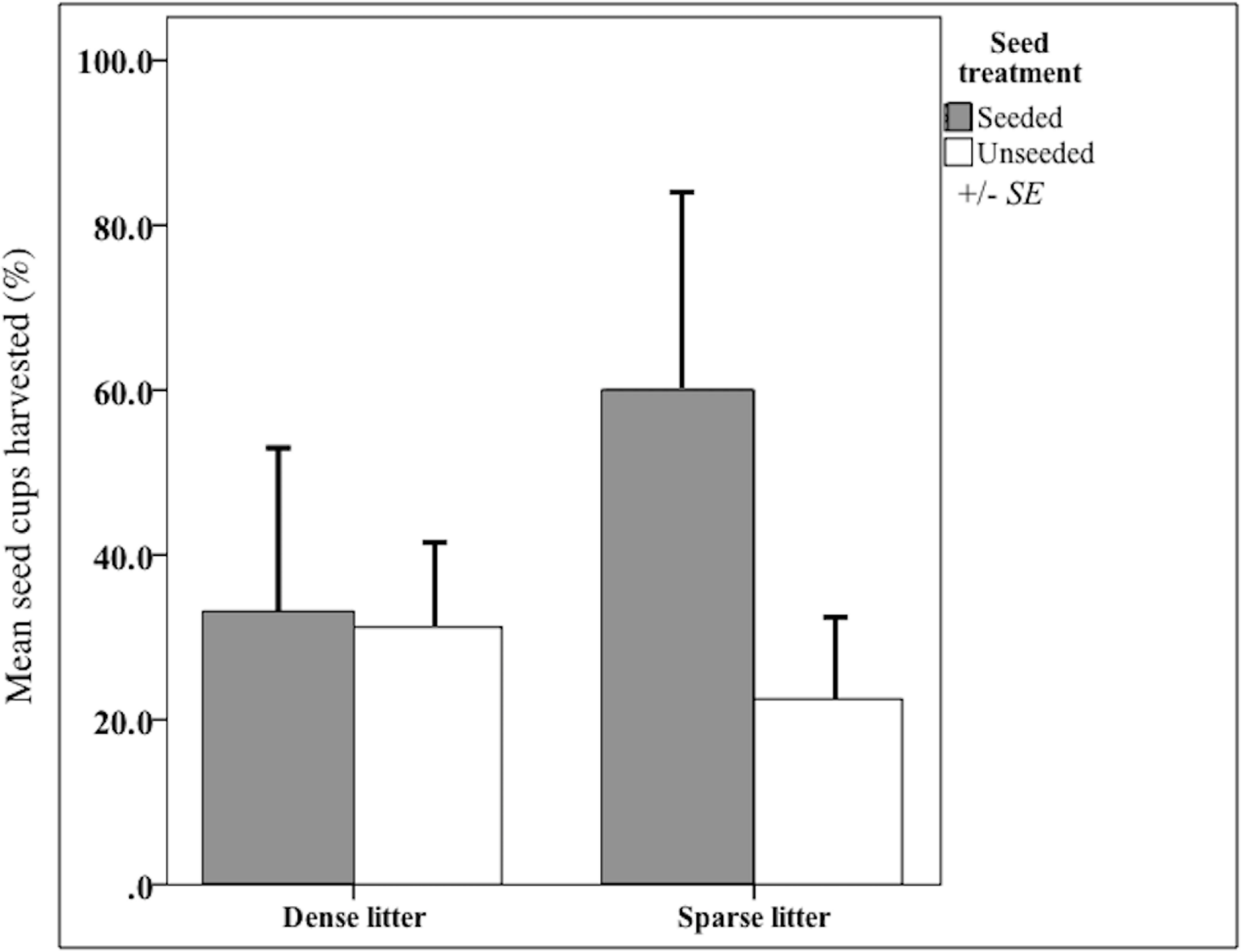
Harvest from seed cups at litter and seed treatments. Mean (SE of the mean) seed-harvested cups (%) at sparse and dense litter treatments where plots were seeded or unseeded, Sevilleta National Wildlife Refuge, New Mexico, USA. Cups presented for 20 days beginning 16 October 2015. *n* = 14 cups in seeded and 29 cups in unseeded plots.

I validated that sown propagules were collected by rodents. Propagules were harvested from nearly 50% of seed clump cups after 20 days (Fig. 2), indicating rodents could find sown seed clumps. When found, a mean of 43% propagules (range = 25-100%, *n* = 3,300 seeds) were collected out of a cup; thus sown seeds could have been harvested and subsequently stored in rodents’ nests, cached or eaten. There were other seed predators observed that also could have removed sown seeds, notably harvester ants (*Pogonomyrmex*) and overwintering flocks of song birds. However, temperatures were too low for ant foragers, thus they were rarely observed. Bird distribution is unpredictable and patchy throughout the fall and winter, causing their probable rate of seed intakes to be locally high but widely dispersed.

### Litter treatments

Structure of the rodent community varied among litter treatments. In sparse plots, the most abundant species was kangaroo rats and in dense plots it was pocket mice (Kangaroo rats and Pocket mice × Litter levels Interaction *F*_1, 8_ = 9.11, *p* = 0.006; Table 1). Dense litter plots had seven species while sparse litter plots had four.

**Figure 3:**
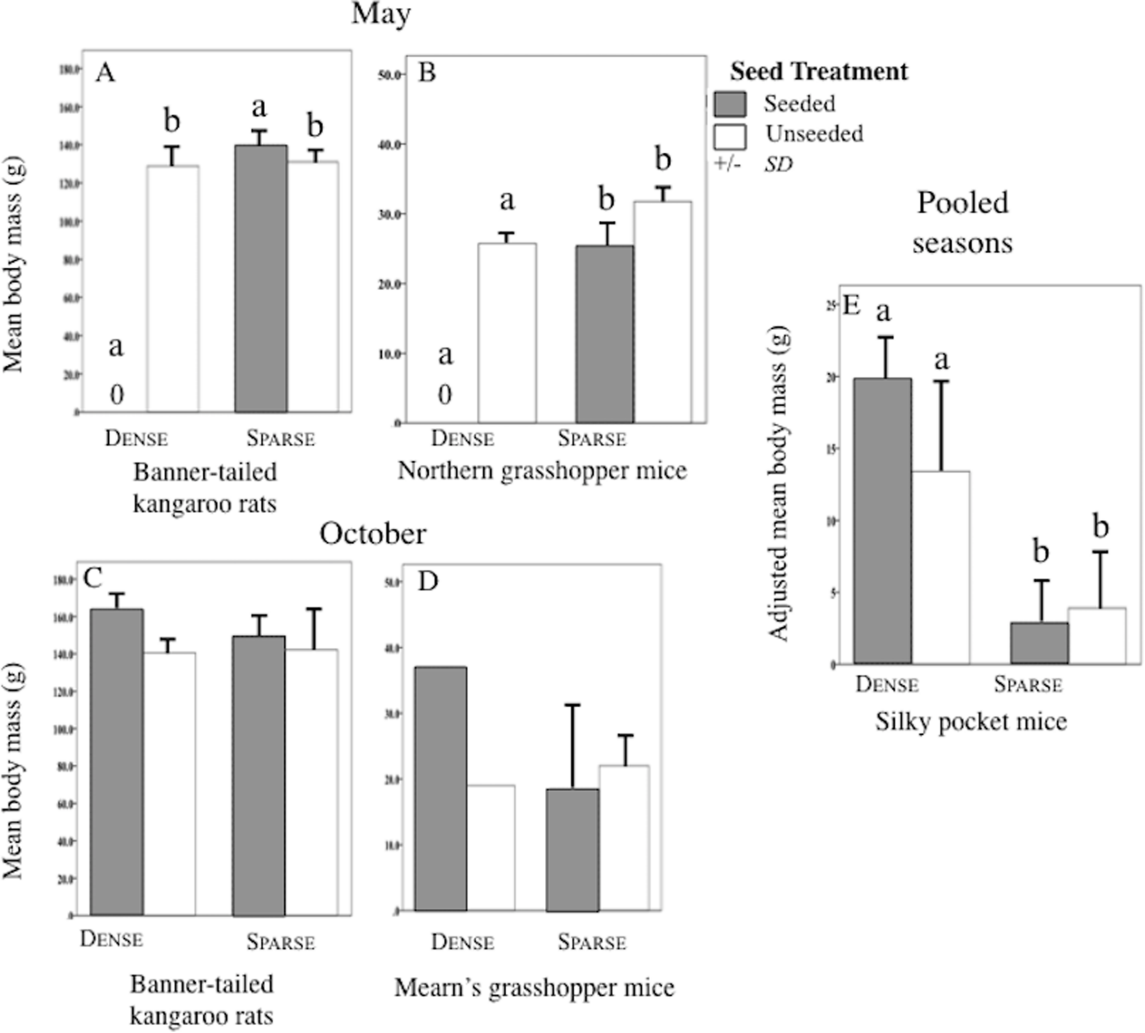
Body mass of rodent species in litter and seed treatments. Mean (SD) body mass (g) of banner-tailed kangaroo rats (A and C), northern grasshopper mice (B), Mearn’s grasshopper mice (D), and adjusted body mass (g) of silky pocket mice (E) at sparse and dense litter treatments where plots were seeded or unseeded, Sevilleta National Wildlife Refuge, New Mexico, USA. Data collected 5–7, 11–13 May 2016 and 27–29 October, 3, 7, and 8 November 2016. Means with different letters are significantly different. Bars with no lines are SD = 0. *n* = 2 plots per treatment combination. Note: Taxa vary in scale.

**Figure 4:**
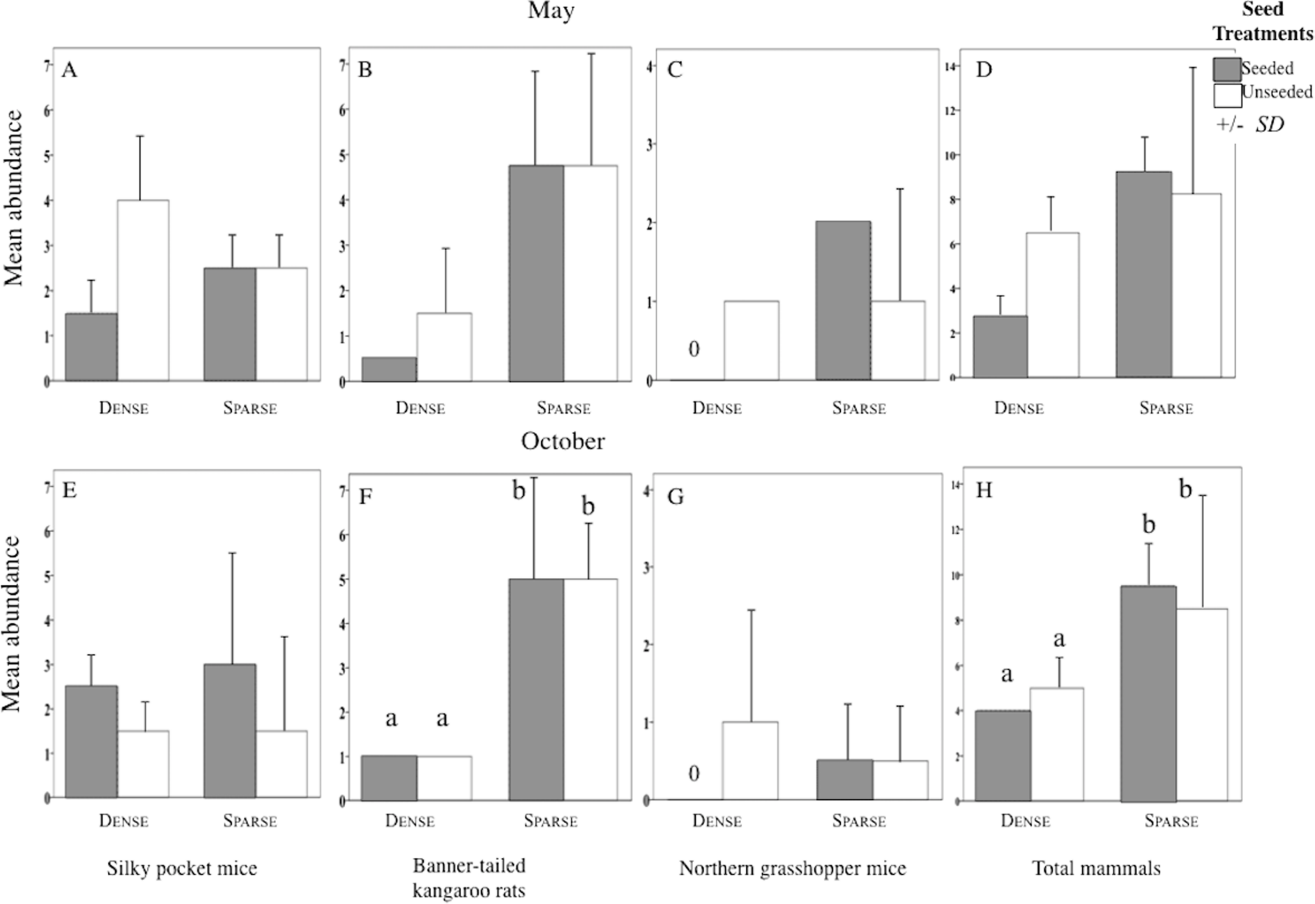
Abundance of each rodent species in litter and seed treatments. Mean (SD) abundance in 0.5-ha plots of silky pocket mice (A and E), banner-tailed kangaroo rats (B and F), northern grasshopper mice (C and G), and total small-mammals (D and H) at sparse and dense litter treatments where plots were seeded or unseeded, Sevilleta National Wildlife Refuge, New Mexico, USA. Data collected 5–7, 11—13 May 2016 and 27–29 October, 3, 7, and 8 November 2016. Means with different letters are significantly different. Bars with no lines are SD = 0. *n* = 2 plots per treatment combination. Note: Taxa vary in scale.

Sparse litter plots had greater mean body weights in May compared to dense litter plots. Mean adult body mass of northern grasshopper mice (Fig. 3B) and mean rodent biomass (sparse plots 224.1 g/ha^−1^, SD 481.0, *n* = 35 rodents; dense plots 68.9 g/ha^−1^, SD 139.2, *n* = 19 rodents) were almost significantly greater in the sparse litter treatment than dense litter (Table 2). In October, the sparse litter treatment had higher mean kangaroo rats abundance (Fig. 4F) and higher mean rodent total abundance (Fig. 4H) compared to the dense litter treatment (Table 3). There were more rodent total offspring captured in the sparse litter treatment (6 offspring) compared to the dense litter treatment (0 offspring; Fishers’s Exact Test χ^2^_1,55_ = 3.28, *p* = 0.08).

**Table 2:** ANOVA table of rodent body masses in litter and seed treatments. Summary of ANOVA results for adult body mass of banner-tailed kangaroo rats, northern grasshopper mice, adjusted body mass of silky pocket mice, and total biomass of nocturnal rodents at sparse and dense litter treatments where plots were seeded or unseeded during cool-season and combined seasons, Sevilleta National Wildlife Refuge, New Mexico, USA. Pocket mouse analysis included the covariate plant litter biomass.

Trapping period & species	Main effects	*F*	*p*-level
May			
Banner-tailed kangaroo rats	seed	4.88	0.04
	litter	0.13	0.72
	seed × litter	2.00	0.13
Northern grasshopper mice	seed	6.60	0.08
	litter	7.74	0.07
Total rodent biomass^a^	seed	3.25	0.09
	litter	3.40	0.08
	seed × litter	1.70	0.21
Pooled seasons			
Adjusted silky pocket mice	seed	1.21	0.28
	litter	4.26	0.05
	seed × litter	2.23	0.15
	covariate	4.69	0.04

**Note:**

a log transformation of data

**Table 3:** ANOVA table of rodent abundance in litter and seed treatments. Summary of ANOVA results for numbers of banner-tailed kangaroo rats, northern grasshopper mice, and total nocturnal rodents at sparse and dense litter treatments where plots were seeded or unseeded during cool- and warm-seasons, Sevilleta National Wildlife Refuge, New Mexico, USA.

Trapping period & species	Main effects	*F*	*p*-level
May			
Northern grasshopper mice^b^	seed	0.17	0.70
	litter	2.52	0.19
	seed × litter	5.83	0.07
October			
Total rodents^a^	seed	0.001	0.98
	litter	7.23	0.04
Banner-tailed kangaroo rats^c^	seed	0.00	1.00
	litter	4.29	0.04

**Notes:**

a Log transformation of data.

b Square root transformation of data.

c Kruskal–Wallis analysis.

In contrast, mean northern grasshopper mice abundance decreased over 60% in sparse litter plots between May and October, whereas in dense litter their abundance remained the same (Figs. 4C and 4G). After adjusting for plant litter biomass, sparse plots had significantly less mean adult pocket mice body mass than dense plots when pooling seasons (Fig. 3E; Table 2). Pocket mice data were pooled among seasons because body mass was similar in each case and it was unlikely I would capture the same individual in May and October—the likelihood of recaptures was just 16%. Mearn’s grasshopper mice population characteristics were unvarying among litter and seed treatments.

### Seed treatments

Seed augmentation influenced population characteristics only during May captures. Average abundance of northern grasshopper mice had a marginally significant interaction (Table 3). In seeded plots they had higher numbers in the sparse litter treatment compared to dense, but in unseeded plots numbers were similar between litter treatments (Fig. 4C). Mean adult body mass of kangaroo rats was significantly greater in the seeded compared to the unseeded treatment (Fig. 3A; Table 2). Between May and October mean pocket mice abundance remained unchanged in seeded plots, but in unseeded plots there was a 50% decline (Figs. 4A and 4E).

Due to one small, lactating female, seeded plots had marginally less mean adult body mass of northern grasshopper mice compared to unseeded plots (Fig. 3B; Table 2). After combining seasons, 20% of pocket mice males were reproductive in seeded plots, whereas in unseeded 100% of the males were reproductive (Fisher’s Exact Test χ^2^_1,10_ = 6.67, *p* = 0.02). Pocket mice data were pooled among seasons because results were similar.

## Discussion

In semiarid grasslands of the Sevilleta National Wildlife Refuge, litter and seed treatments differentially modified population characteristics of nocturnal rodents. Their higher characteristics in sparse litter demonstrated that litter can be important to this small-mammal community in 1 year. Seed supplements generally enhanced characteristics in May indicating that seeds can be a limiting resource for rodents winter through spring. Examining each species suggests that litter level has a modestly stronger influence than seed level in the short term. In a similar desert study, the distribution of dusky hopping-mice was most strongly influenced by the amount of habitat and secondly by levels of food (Allen et al., 2018). Managing for a mosaic of differing litter levels could provide improved resources for this rodent community.

In the sparse litter treatment, total rodent abundance, rodent biomass, and offspring numbers were higher than in the dense litter treatment supporting the first hypothesis. Generally, species associated with open, low litter habitats were most influenced by the sparse litter treatment. These results are consistent with other litter manipulation studies (Abramsky, 1978; Thompson & Gese, 2013) and with observations in black-tailed prairie dog towns where litter is significantly lower than outside prairie dog towns (Agnew, Uresk & Hansen, 1986). During my study, few offspring were captured, thus higher abundance of total rodents is likely attributable to adult higher survival from spring to fall, immigration into the plots, or both. For example, as Price (1978) manipulated the amount of open and dense desert microhabitats, heteromyids moved to the landscape with the greatest expanse of their preferred microhabitat. Additionally, abundance of banner-tailed kangaroo rats can increase in recently manipulated, preferred habitat due to adult survivorship (Waser & Ayers, 2003) and immigration (Cosentino et al., 2014; Waser & Ayers, 2003). In future studies, trapping more frequently may uncover offspring numbers. Greater biomass of rodents was due to their abundance-biomass relationship decoupling between the large-bodied kangaroo rat’s numbers and the smaller alteration from the numerically dominant, small-bodied pocket mice. Additionally, northern grasshopper mice were heavier in the sparse litter treatment thus contributing to community rodent biomass.

Dense litter levels have strongly negative influence on abundance and body mass of northern grasshopper mice; a result supported by prior studies (Abramsky, 1978; McCarty, 1978; Thompson & Gese, 2013). The cause appears to be thickness rather than cover because grasshopper mice were not caught in the high litter biomass plots, but instead were recorded in the remaining dense cover plots with their 36% lower litter biomass. Abramsky (1978) also found northern grasshopper mice were absent where litter biomass was 100 g/m^2^ higher than my measurements. Stapp (1997b) concluded that dense litter biomass could inhibit access to insect prey. My results indicate access to seeds may also be inhibited. Northern grasshopper mice are rarely caught in grasslands at Sevilleta NWR probably because litter is thick where there are few disturbances ((Newsome, 2017) Five-Points grassland, 1989–2012).

In contrast, higher body mass of pocket mice was more commonly found in dense litter plots than sparse, thus not supporting the first hypothesis. Differences may be attributed to their need to forage nightly (Best & Skupski, 1994) in conjunction with perceived predation risk. Even though it is probably more difficult for pocket mice to maneuver through dense litter to harvest food, this could be offset by the ability to spend more time foraging in dense litter plots than in sparse litter. Similarly, Lemen & Rosenzweig (1978) found that pocket mice collect more seeds in 0.6 m high grasses compared to bare ground. Pocket mice may also have used torpor to save energy in sparse litter plots and torpor leads to loss in their body mass (Best & Skupski, 1994). Unexpectedly, their abundance was similar between litter treatments. These results do not support the null hypothesis; instead, abundance may be altered by unknown factors.

Seeded plot effects occurred during spring sampling but disappeared during the fall even though there had been two more additions of sown seeds during the dry warm-season. Trapping every 2 months may reinforce these results or reveal a short-term pattern. Seed supplementation indirectly increased body mass of kangaroo rats and abundance of northern grasshopper mice, reflecting the use of stored seeds. Seeds sown the previous fall were collected and stored by kangaroo rats during the same season when they store the highest number of seeds (Best, 1988; Hoffmeister, 1986), thus promoting their body mass growth during the cool-season. These results concur with Brown & Munger (1985) who concluded that higher mean body mass was partially the result of growth. Unexpectedly, northern grasshopper mice had higher numbers in seeded compared to unseeded plots; the result is contrary to prior supplementation studies (Abramsky, 1978; Brown & Munger, 1985). Since northern grasshopper mice mostly collect seeds from fall through early spring (Flake, 1973; Hope & Parmenter, 2007), their abundance was probably indirectly caused by consuming cached, sown seeds when arthropods were low which may have occurred during the dry cool-season (Jahoda, 1970; McCarty, 1978). And, as with total rodents, increased spring abundance suggests adult immigrate into plots with additional seeds during the previous fall.

The abundance of pocket mice was similar between seed treatments, supporting my third hypotheses. With the poor recapture estimates, however, these results should be viewed with caution. Still, similar results are reported following supplemented seeds (Brown & Munger, 1985) and higher herbaceous seed production (Whitford, 1976). From May to October their abundance was stable in seeded plots while abundance decreased in the unseeded plots, corresponding to a prior study in desert-dwelling pocket mice (*Chaetodipus*) (Orland & Kelt, 2007). The stable numbers may have been influenced by seed augmentation during the warm-season drought since this diminutive species needs to forage nightly. The frequency of reproducing males was lower in seeded plots suggesting that dominant reproductive males may have tolerated other males more readily when supplemented seeds were available.

Additional factors may have shaped rodent population characteristics including predation (Shenbrot, 2014), competition (Genoways & Brown, 1993; Heske, Brown & Mistry, 1994; Lima et al., 2008), and smaller sizes of home ranges when resources become plentiful (Boutin, 1990). Negative correlations between species hint an interaction might be influencing the results. But the abundance of the predator northern grasshopper mice (McCarty, 1978; Punzo, 2007; Stapp, 1997a) was uncorrelated with abundances of banner-tailed kangaroo rats and silky pocket mice. Banner-tailed kangaroo rats, a known competitor (Bowers, Thompson & Brown, 1987; Heske, Brown & Mistry, 1994), was uncorrelated with silky pocket mice and Mearn’s grasshopper mice. However, northern grasshopper mice exclude southern grasshopper mice (*O. torridus*) from suitable habitat (Gennaro, 1968); an outcome that may extend to the closely related Mearn’s grasshopper mouse, possibly eliminating captures in May.

My study, like most litter investigations in the field, used litter reduction methods that hampered our ability to establish causality or determine mechanisms without confounding factors from the method employed. Therefore, I attempted to reduce any confounding factors in two ways: I chose to mimic fire, a naturally occurring disturbance, because the plant and animal communities have adapted. I began sampling 1 year after the prescribed burn to allow some recovery of community structure and function. Specifically, directly after fire in semiarid grasslands, there is a decrease in predator cover when above-ground, live plant biomass is decreased (Brockway, Gatewood & Paris, 2002; Scheintaub et al., 2009). As a result, some grasses and perennial dicot productivity (Ladwig et al., 2014; Parmenter, 2008), diversity and density (Collins et al., 2017; Nicolai, 2019) may all increase. By the following year, there can be substantial recovery of aerial vegetation cover. Further, in a prior study of prairie arthropods, this important food source was decreased by a spring prescribed burn, but by summer, abundance and richness had generally recovered (Harper et al., 2000). The majority of available seeds in the soil seed bank was probably unaltered by fire even if surface seeds and rodent caches were lost. For example, perennial grasses re-establish themselves after burning, partially because seedlings recolonize from a soil seed bank (Eriksson, 1989).

One year of rodent sampling did not test the dynamics of this rodent community’s populations. Therefore, rodent responses should be viewed as short-term. If I had prolonged the study into another year, the amount of litter accumulated would add a further complication to interpretation of the litter treatment results. A grazing method such as reintroducing prairie dogs, which leaves litter fairly similar among years, may be a better option for longterm studies of population dynamics. Despite these limitations, I believe this study reveals the potential importance of litter’s physical architecture on rodents when examining the consequences of disturbance suppression in semiarid grasslands.

## Conclusions

I investigated the short-term responses of nocturnal rodents from simultaneously varying two litter cover levels and seed densities. My results demonstrate that litter’s physical architecture is equally as important as food pulses on abundances and body masses during 1 year in the semiarid grassland at Sevilleta NWR. Specifically, total rodent abundance, rodent biomass, and offspring numbers were higher in the dense litter treatment than in the sparse litter treatment, indicating that foragers could gather food more efficiently, and for banner-tailed kangaroo rats, they could also detect and escape predators. However, greater body mass of pocket mice was found in dense litter plots compared to sparse. In the spring, seed supplementation increased body mass of kangaroo rats and abundance of northern grasshopper mice compared to unseeded plots, reflecting the use of stored seeds during the winter. My results suggest that litter cover presents varying levels of foraging efficiency and predation risk to each species. Managers might benefit population characteristics of nocturnal, grassland rodents by manipulating and providing a mosaic of varying levels of plant litter through the use of litter-reducing methods such as reintroducing prairie dogs and conducting prescribed burns.

## Supplemental Information

10.7717/peerj.9465/supp-1 Supplemental Information 1 Raw Trapping Data. The date, species, sex, reproductive condition, weight, age, treatment, and plot where individuals were captured.

## Additional Information and Declarations

### Data Availability

The following information was supplied regarding data availability: The raw trapping data are available in the Supplemental File. Data from the U.S. Fish and Wildlife Service can be requested here: https://fws.gov/refuge/Sevilleta/visit/plan_your_visit/contact_us.html or by contacting Jon Erz at Sevilleta National Wildlife Refuge, P.O. Box 1248, Socorro, NM 87801 USA.
